# THE POWER OF RURAL PARTNERSHIP: MEETING NEEDS AND FOSTERING INNOVATION THROUGH THE WYOMING GWEP

**DOI:** 10.1093/geroni/igac059.885

**Published:** 2022-12-20

**Authors:** Christine McKibbin, Catherine Carrico

## Abstract

The challenges of supporting the health and social needs of older adults and caregivers in rural and frontier areas are well-documented. It is common for rural older adults to experience barriers in accessing geriatric specialists, care coordination services, and caregiver support and education programs. The Wyoming Geriatric Workforce Enhancement Program (WyGWEP) is an innovative partnership comprising an academic geriatrics program, primary care delivery sites, single-unit on aging representing community-based organizations, and a tribal health program. This partnership, funded by the Health Resources and Services Administration, provides the infrastructure to assess needs, provide education and training, create programs to address gaps in care, implement practice innovation, and advocate for needs of rural older adults. This symposium includes five presentations detailing the unique projects of the WyGWEP partnership and the impact of this collaborative work on a variety of stakeholders. The results of a mixed-methods evaluation of the WyGWEP partnership will describe the benefits to the partners and areas for growth. A collaborative effort to assess the needs of older adults informs recommendations to support rural aging in place. Schenck et al. will describe the adaptation of the widespread ECHO model for use with dementia caregivers in rural and remote locations. Representatives from Wyoming’s only Program for All-Inclusive Care of the Elderly (PACE) will explain the cost of a recent decision to de-fund and close this important program. Finally, the impact of a novel Chronic Care Management implementation program will be discussed, including sustainable billing revenue produced by rural primary care clinics.

